# Demographic Associations of Diabetes Status by Both Fasting Plasma Glucose Concentration and Glycated Hemoglobin in a Community Survey in Galle District, Sri Lanka

**DOI:** 10.1155/2020/6127432

**Published:** 2020-04-06

**Authors:** Keddagoda Gamage Piyumi Wasana, Anoja Priyadarshani Attanayake, Thilak Priyantha Weerarathna, Kamani Ayoma Perera Wijewardana Jayatilaka

**Affiliations:** ^1^Department of Biochemistry, Faculty of Medicine, University of Ruhuna, Galle, Sri Lanka; ^2^Department of Medicine, Faculty of Medicine, University of Ruhuna, Galle, Sri Lanka

## Abstract

Diagnostic tools used in detecting individuals with diabetes mellitus (DM) include fasting plasma glucose (FPG), glycated hemoglobin (HbA_1C_), and oral glucose tolerance test (OGTT). The present study was aimed to determine the demographic associations of diabetes status by both tests (FPG and HbA_1C_) in Galle district, Sri Lanka. 147 adults (30–60 years) who are having FPG ≥ 126 mg/dL underwent demographic evaluations and testing for HbA_1C_. Group 01 (diabetes status diagnosed by both tests) and group 2 (diabetes status diagnosed only by FPG) were compared using independant sample *t*-test and chi-square test. Logistic regression was used to study the association between the demographic factors and the diabetes status by both tests. Of the 147 study subjects, 38.1% were males, 61.9% were females, and 63.3% had a family history of diabetes among first-degree relatives (FDR). Mean age, body mass index (BMI), waist circumference (WC), FPG, and HbA_1C_ of the participants were 48.4 ± 7.2 years, 25.1 ± 4.0 kg/m^2^, 88.8 ± 9.0 cm, 139.4 ± 30.1 mg/dL, and 6.4 ± 0.7%, respectively. The prevalence of diabetes based on both tests was 55.1%. There is a significant difference in mean BMI and WC while no significant differences in mean age between groups 01 and 02. No association was seen between gender and diabetes status (*X*^2^(1) = 0.086, *p*=0.770), while a significant difference was observed between DM among FDR and diabetes status (*X*^2^(1) = 33.215, *p* < 0.001). Significance of odds of having diabetes by both tests with rising BMI (OR = 1.97, CI 1.15–3.36, *p*=0.013) and DM among FDR (OR = 7.95, CI 3.54–17.88, *p*=0.000) was seen. We conclude rising BMI and having DM among FDR are strongly associated with diabetes status diagnosed by both tests of FPG and HbA_1C_ in community screening.

## 1. Introduction

Diabetes mellitus (DM) is a leading metabolic disorder with rising incidence worldwide. According to the International Diabetes Federation (IDF) data, 415 million adults suffer from DM at present, and this number will be 642 million by 2040 [1]. Importantly, the low- and middle-income countries are vulnerable to DM and its associated complications. Notably, the prevalence of DM has increased as an epidemic in the South-East Asian region in the past few years. The IDF has estimated that 6.8–10.8% of the adult population is suffering from DM in the South-East Asian countries [1]. The prevalence of DM in Sri Lanka, a developing South Asian country, was 7.9% in 2016 [2]. The early diagnosis of DM is very important as it offers a worthy opportunity to prevent its associated complications. According to the latest guidelines laid down by the American Diabetes Association (ADA), the screening for DM should be performed in asymptomatic adults aging >45 years and repeated in every three years. Overweight individuals and those with risk factors for diabetes should be screened earlier than 45 years [3].

Different diagnostic tools such as fasting plasma glucose (FPG), glycated hemoglobin (HbA_1C_), and oral glucose tolerance test (OGTT) are used in the diagnosis of DM. According to the ADA guidelines, the individuals with FPG concentration ≥126 mg/dL, HbA_1C_ level ≥6.5%, and 2 hour plasma glucose value after 75 grams OGTT ≥200 mg/dL are considered as having DM [4]. Although most community surveys use FPG to diagnose individuals with diabetes, its sensitivity and specificity vary [5]. Recently, estimation of HbA_1C_ has been recommended for the purpose of diagnosis of abnormalities in glucose tolerance including prediabetes and diabetes. HbA_1C_ test has several advantages over the FPG. These include good biological stability at ambient temperature, ability to use in the nonfasting state, and allowing the test to be performed at any time of the day [6]. HbA_1C_ test indicates a stable index of long time glycemic load which is related to three months of average glucose concentration in plasma [7]. However, due to lack of standardization facilities, relative unavailability, and high cost of HbA_1C_ testing, the FPG has been the preferred and widely used test over HbA1C test in the diagnosis of DM [8]. Moreover, several biological factors such as clinical conditions that alter erythropoiesis, glycation rate, erythrocyte destruction, and analytical interferences such as hyperbilirubinaemia, carbamylated hemoglobin, certain medications, and hemoglobin variants affect for the alteration cutoff values of HbA_1C_ [9]. Although the gold standard of diagnosis of DM is OGTT, its wider use in the community setting is hindered by many practical issues such as preparation of patients, cumbersome procedure, and poor reproducibility [8].

To date, the demographic associations of diabetes status by combination of both FPG and HbA_1C_ have not been explored in Sri Lanka. Therefore, the present study was aimed to determine the demographic associations of diabetes status by both FPG and HbA_1C_ in a community survey in Galle district, Sri Lanka.

## 2. Method

### 2.1. Ethical Consideration

Ethical clearance was granted from Ethical Review Committee, Faculty of Medicine, University of Ruhuna, Sri Lanka (14.06.2017:3.9).

### 2.2. Data Collection

The present study was a community-based, cross-sectional study carried out in the Galle district, Southern province, Sri Lanka, by involving a total of 147 newly diagnosed patients between the age of 30 and 60 years. The sample frame of the study was designed using 19 divisional secretariats in Galle district. All participants were invited to visit the Faculty of Medicine, University of Ruhuna, Sri Lanka, before 8.30 a.m. after an overnight fasting of 8–10 hours. Venous blood samples were collected from all recruited subjects for the determination of FPG concentration and HbA_1C._ FPG concentration was determined by an enzymatic (glucose oxidase) colorimetric method. The drawing of blood samples was performed by qualified laboratory technicians using the standard protocols. The percentage of HbA_1C_ was estimated in study subjects spectrophotometrically using a resin exchange method. Before drawing of blood samples, written consent was obtained from each participant. The individuals with FPG concentration ≥126 mg/dL and HbA_1C_ ≥6.3% were considered as diagnosed patients with type 2 diabetes mellitus (T2DM). The demographic data including age, gender, height, weight, waist circumference (WC), and self-reported family history of diabetes among the first-degree relatives were obtained. Weight was recorded to nearest 0.5 kg, and height was measured to the position without shoes by using a height bar [10]. WC was recorded using a flexible measuring tape. Body mass index (BMI) of study subjects was calculated as weight (kg) divided by height squared (m^2^). Patients who are suffering from complaints as renal, liver, cardiac, respiratory diseases, other chronic or acute diseases, thyroid disorder, psychiatric problems, and pregnant mothers were excluded from the study. Patients who are using antidiabetic or antilipidemic drugs were also excluded.

### 2.3. Statistical Analysis

All data were analyzed using SPSS software. The continuous variables were presented as mean ± SD, while the categorical data were expressed as percentages. After having checked the normality of the variables of age, BMI, and WC in group 1 (diabetes status diagnosed by both tests of FPG and HbA_1C_) and group 2 (diabetes status diagnosed only by FPG) by using Kolmogorov–Smirnov test, independent sample *t*-test was used to compare both groups. Relationship between categorical data was assessed using chi-square test. Binary logistic regression was used to study the association between the demographic factors (independent variables) and the diabetes status by both FPG ≥ 126 mg/dL and HbA_1C_ ≥ 6.3% (dependent variable). *p* ≤ 0.05 was considered as statistically significant.

## 3. Results

Of the 147 study subjects, 56 (38.1%) were males and 91 (61.9%) were females. From all the subjects, 93 subjects (63.3%) had a family history of diabetes among the first degree of relatives.

Mean age of the study subjects was 48.4 ± 7.2 years, mean BMI was 25.1 ± 4.0 kg/m^2^, and mean WC was 88.8 ± 9.0 cm. Mean FPG and HbA_1C_ were 139.4 ± 30.1 mg/dL and 6.4 ± 0.7%, respectively (Table 1).

**Figure 1:**
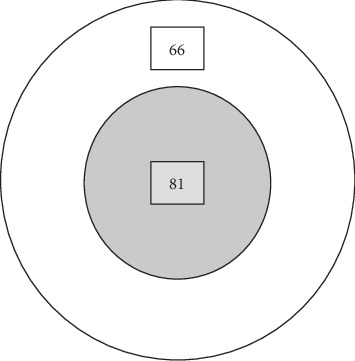
DM was diagnosed by both tests of FPG and HbA_1C_ (gradient circle). DM was diagnosed only by FPG (rest of the figure).

Among all the subjects of 147 detected to have diabetes based on FPG, only 81 subjects were found to have diabetes by HbA_1C_ (Figure 1). The prevalence of diabetes based on both tests of FPG and HbA_1C_ was 55.1%.

Results of the independent sample *t*-test showed that there was a significant difference in mean values of BMI and WC between group 01 and group 02. Furthermore, the results showed that there is no significant difference in mean values of age between the two groups (Table 2).

Results of the chi-square test showed that there is no association between gender and diabetes status (*X*^2^(1) = 0.086, *p*=0.77). There is a significant difference between DM present in first-degree relatives and diabetes status (*X*^2^(1) = 33.215, *p* < 0.001). Regression analysis revealed significant of odds of having diabetes status by both tests (FPG and HbA_1C_) with rising BMI (OR = 1.96, CI 1.15–3.36, *p*=0.01) and family history among first-degree relatives of diabetes (OR = 7.95, CI 3.54–17.88, *p* < 0.001) (Table 3).

## 4. Discussion

Apart from the biochemical parameters used in the diagnosis of DM such as FPG, HbA_1C_, and OGTT, risk factors associated with diabetes are also important in the screening for DM of patients. Diabetes risk factors such as general obesity, central obesity, age, and DM among first-degree relatives were considered as demographic factors in the present study. General obesity is measured by BMI, while the central obesity is measured by WC. A population-based cohort study conducted in central Iran involving 1,765 participants has revealed that general obesity and central obesity are significant risk factors for DM [11]. Increase in weight is positively associated with an increase in glucose concentration in blood, and hence, it increases the risk of developing DM [12]. Even though diabetes among first-degree relatives is a risk factor for T2DM, the inheritance pattern is still unknown. Associations of selected risk factors with DM status diagnosed by one or more diagnostic tools are well established [13, 14]. These diagnosis tools possess significant limitations when the tools are performed alone. Determination of diabetes status by combination of FPG and HbA_1C_ provides several advantages such as better sensitivity, reliability, specificity, greater preanalytical stability, and lower day-to-day perturbation of blood glucose caused by acute events like stress or illness [9]. The present study was focused to determine the demographic associations of diabetes status diagnosed by both FPG and percentage of HbA_1C_ in a selected community in southern Sri Lanka.

All subjects in this study were newly diagnosed patients with DM with a FPG concentration ≥126 mg/dL. HbA_1C_ test was performed in parallel to verify whether all subjects are diagnosed with DM or not based on the % HbA_1C_ results. Even though ADA which recommended cutoff point for HbA_1C_ test is ≥6.5%, in the present study, we used HbA_1c_ ≥6.3% as the cutoff level based on the results of a local study conducted in Sri Lanka recently [15].

From the total number of 147 subjects with FPG ≥126 mg/dL, only 81 subjects (55.1%) were diagnosed with diabetes based on the HbA_1C_ test results. The results suggest that FPG and HbA_1C_ have not corroborated similar performance as diagnostic tools for diabetes. Even though HbA_1C_ test is an alternative screening tool to FPG for Asian population [9], the finding of the present study clearly indicated that HbA_1C_ test could not be used as an alternative diagnosis tool to FPG for the diagnosis of DM in Sri Lankan setting. Similar observations had been made in the previous studies carried out in Sri Lanka [15]. A study conducted in a Taiwan population has also reported that more patients could be diagnosed with DM using HbA_1C_ over the FPG concentration [16].

The present study revealed that the individuals who are diagnosed by FPG followed by HbA_1C_ have significantly high BMI (*p*=0.02), WC (*p*=0.03), and DM among first-degree relatives (*p* < 0.001) compared with individuals who are diagnosed by FPG alone. Therefore, it is better to use the diagnosis tool of FPG followed by HbA_1C_ test to confirm the DM status for the individuals who are having high BMI, WC values, and DM among first-degree relatives. Furthermore, findings of the study revealed that the demographic factors including BMI (*p*=0.01) and DM among first-degree relatives (*p* < 0.001) are significantly associated with the diabetes status diagnosed by both tests of FPG and HbA_1C._ Therefore, diabetes risk factors which confer long-term impact on glycaemia such as genetic predisposition and increasing BMI are associated with diabetes status diagnosed by both FPG and HbA_1C._. Other risk factors such as acute stress, consumption of high carbohydrate diet, or refined sugars are more likely to affect diabetes status diagnosed by FPG alone. This is the first study that investigated demographic associations of diabetes status diagnosed by a combination of FPG and HbA_1C_ in a population of Sri Lanka.

The main strength of this study is that the inclusion of previously healthy and newly diagnosed individuals with diabetes. Small sample size and not performing OGTT in selected participants are the main limitations of this study. As the study sample was selected from Galle district in Sri Lanka, this sample is not representing the total population in Sri Lanka. Therefore, it is important to carry out the same study among different population groups in different areas in Sri Lanka to confirm whether the results of the present study are applicable to all Sri Lankans. Furthermore, information regarding the lifestyle factors and other factors such as physical activity, smoking, using of alcohol, diagnosis of polycystic ovarian syndrome, and history of gestational diabetes mellitus was not inquired of the study subjects in the present study.

In conclusion, FPG and HbA_1C_ tests differ in their diagnostic performances as diabetes diagnostic tools among Sri Lankans. Furthermore, it is important to perform FPG followed by HbA_1C_ test to diagnose and confirm the diabetes status for the individuals who are having high BMI and WC values. Rising BMI and having DM among the first-degree relatives are strongly associated with diabetes status diagnosed by both FPG and HbA_1C_.

## Figures and Tables

**Table 1:** Descriptive statistics of study subjects.

	Mean	SD
Age (years)	48.4	7.2
BMI (kg/m^2^)	25.1	4.0
WC (cm)	88.8	9.0
FPG (mg/dL)	139.4	30.1
HbA_1C_ (%)	6.4	0.7

BMI: body mass index, WC: waist circumference, FPG: fasting plasma glucose, and HbA_1C_: glycated hemoglobin.

**Table 2:** Age, BMI, and WC of study subjects in group 01 and 02.

	Group 01 (mean)	Group 02 (mean)	*p* value
Age (years)	48.31	48.55	0.84
BMI (kg/m^2^)	25.83	24.31	0.02
WC (cm)	90.25	87.06	0.03

BMI: body mass index; WC: waist circumference.

**Table 3:** Results of binary logistic regression analysis for the demographic associations of diabetes status by both FPG and HbA_1C_.

Variable	OR	95% CI	*p* value
Gender	1.01	0.45–2.28	0.96
Age	0.99	0.94–1.05	0.88
BMI	1.96	1.15–3.36	0.01
WC	1.00	0.95–1.05	0.99
DM among first-degree relatives	7.95	3.54–17.88	<0.001

BMI: body mass index, WC: waist circumference, and DM: diabetes mellitus.

## Data Availability

The data used to support the findings of this study are included within the article, and the raw data are also available upon request.
